# Microbotanical Evidence of Domestic Cereals in Africa 7000 Years Ago

**DOI:** 10.1371/journal.pone.0110177

**Published:** 2014-10-22

**Authors:** Marco Madella, Juan José García-Granero, Welmoed A. Out, Philippa Ryan, Donatella Usai

**Affiliations:** 1 Complexity and Socio-Ecological Dynamics Research Group (CaSEs), Barcelona, Spain; 2 ICREA – Department of Humanities, Universitat Pompeu Fabra (UPF), Barcelona, Spain; 3 Department of Archaeology and Anthropology, Institució Milà i Fontanals - Spanish National Research Council (CSIC), Barcelona, Spain; 4 Graduate School Human Development in Landscapes, Christian-Albrechts-Universität, Kiel, Germany; 5 Department of Conservation and Scientific Research, The British Museum, London, United Kingdom; 6 Institute of Archaeology, UCL, London, United Kingdom; 7 Centro Studi Sudanesi e Sub-Sahariani, Treviso, Italy; Chinese Academy of Sciences, China

## Abstract

The study of plant exploitation and early use of cereals in Africa has seen over the years a great input from charred and desiccated macrobotanical remains. This paper presents the results of one of the few examples in Africa of microbotanical analyses. Three grave contexts of phytolith-rich deposits and the dental calculus of 20 individuals were analysed from two Neolithic cemeteries in North and Central Sudan. The radiocarbon-dated phytoliths from the burial samples show the presence of Near East domestic cereals in Northern Sudan at least 7000 years ago. Phytoliths also indicate the exploitation of wild, savannah-adapted millets in Central Sudan between 7500 and 6500 years ago. The calculus samples contained starch grains from wheat/barley, pulses and millets, as well as panicoid phytoliths. This evidence shows that Near East domestic cereals were consumed in Northern Africa at least 500 years earlier than previously thought.

## Introduction

Wheat (*Triticum* spp.) and barley (*Hordeum vulgare* L.) were domesticated 10,500 years ago in the Near East, from where they spread east (to Central and South Asia) and west (to Europe and the Mediterranean basin, including North Africa) [1]. The oldest evidence of domesticated cereal crops in North Africa, dated at 4650–4350 cal BC, comes from the Fayum Neolithic at Kom W and K [2].

The Neolithic of Nubia (c. 6000–3500 cal BC) and Central Sudan (5000–3500 cal BC), which appears after non-agricultural, ceramic Mesolithic groups [3], is typically described as centred on pastoralism [4], [5]. While seeds and fruits are occasionally found [6], [7], [8], they are very scarce. This scarcity has been attributed to taphonomy in relation to wet/arid cycles and high microbial activity [9], [10], and sometimes to inadequate archaeobotanical recovery [11]. Possible evidence of plant processing comes from numerous grinding stones from cemeteries. The grinding of tubers and grass seeds has been suggested [7], [12] and phytolith analysis of quern stones from Conical Hill, Wadi Howar (4300 cal BC) in western Sudan, confirms the use of chloridoid and panicoid grasses [13].

The available data on plant remains, therefore, give only restricted information about the processes related to the transition to agriculture. While the period of wild plant gathering during the Mesolithic and the use of crop plants in the later Neolithic are better understood, the introduction of domestic crops (cereals) in this region and the exploitation of wild grains are poorly documented. For the period after the introduction of domesticated crops, it is suggested that the exploitation or cultivation of local (wild) grasses may have continued, at least in the savannah-like regions with a relatively moist climate of Central Sudan [12], [14], [15].

**Figure 1:**
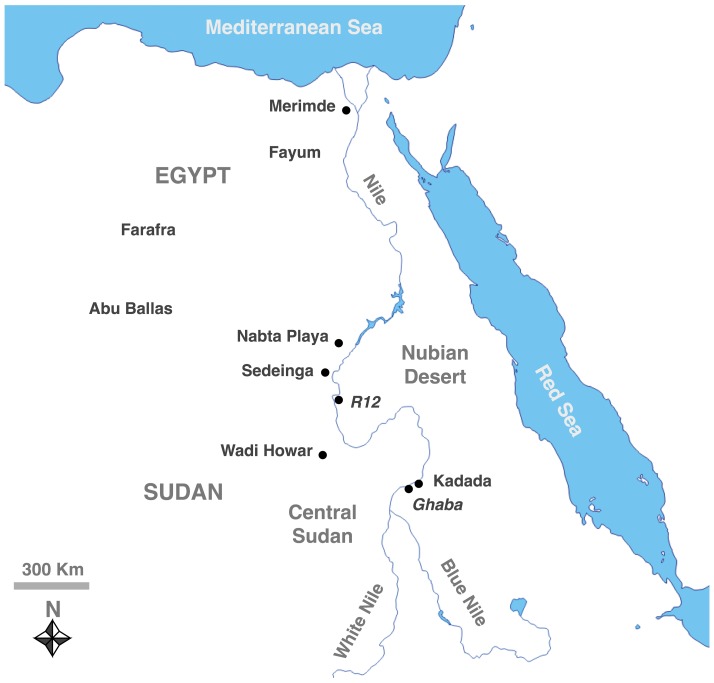
Map showing the location of R12 and Ghaba, as well as other settlements in Egypt and Sudan mentioned in the text.

The evidence emerging from some Neolithic Sudanese cemeteries suggests that plant exploitation played an important role in the supposed pastoral economy [16]. In here we discuss the Central Sudan Early Neolithic cemetery of Ghaba and the Nubian Middle Neolithic cemetery R12 [17] (Fig. 1). We focused our analyses on silica skeletons (phytoliths in anatomical articulation) and starch grains from two complementary contexts: radiocarbon dated pillow-like structures found under the skulls of three individuals (Table 1) and dental calculus from 20 individuals.

**Table 1:** Radiocarbon dates from phytoliths of the pillow-like structures in R12 and Ghaba cemeteries.

Cemetery	Grave	Conventional radiocarbon age	2σ calibrated result (95% probability)	Laboratory number
R12	46	6240±40 years BP	5311–5066 cal BC	Beta-351736
Ghaba	233	6620±40 years BP	5620–5480 cal BC	Beta-359170
Ghaba	295	5800±40 years BP	4730–4540 cal BC	Beta-371517

### Case Study

The R12 cemetery is located on the bank of the Seleim palaeo-channel (in the Dongola Reach in Nubia) that was active between 6200 and 3700 cal BC [18], [19]. The site produced 166 graves containing remains of 198 individuals [20]. Two Middle Neolithic phases were identified: phase A, dated to 5000–4500 cal. BC, and phase B, dated to 4500–4000 cal. BC [20], [21]. Ceramics and animal bones (cattle, sheep, goat and wild animals) are present in both phases [22], but plant macroremains were not recovered. Possible, indirect indications of plant use come from grinding stones encountered in many graves and an unusually large pot from grave 138, which may have been used for storage [23]. Isotope studies of 22 human skeletons from a variety of phases show a mixed isotope signature between C3 and C4 input [24]. This might be directly related to the plant intake or to the consumption of animals grazing on C4 grasses. There is evidence from teeth analysis of tooth abrasion and dental caries, the latter indicating that the intake of food rich in carbohydrates played an important role in the diet [20].

The cemetery of Ghaba, in the Shendi region of Central Sudan, is located on the eastern bank of the Nile, a few hundred meters away from the river. Spontaneous vegetation is nowadays scarce, but the Neolithic climate presumably was more humid and floods occurred more often during the Middle Holocene [25]. Occupation at the cemetery has been dated to 4750–4350 and 4000–3650 cal BC, thus being partly contemporaneous with R12 [3], [26]. The pottery differs from the Nubian one but some common traits point to interregional contacts [17]. Biological remains such as bucrania of domestic cattle, animal bone tools and mollusc shells have been recovered, but no plant macroremains were found.

## Materials and Methods

All necessary permits were obtained for the described study, which complied with all relevant regulations. Excavation and export permits were provided by the National Corporation for Antiquities and Museums (NCAM), Republic of The Sudan, Ministry of Tourism, Antiquities and Wildlife. The project has annual rolling permits for exporting all material to be studied and analysed. Skeletal materials of R12 are held at the British Museum (London, UK) and of Ghaba are held at the Research Centre in Evolutionary Anthropology and Palaeoecology (Liverpool, UK). The sediment materials are held by the project director (Donatella Usai, University of Padova, Italy) and are available upon request. The entire set of analysed samples (20 calculus and 3 sediment microscopy slides) is stored in the collections of the BioGeoPal Laboratory of the University Pompeu Fabra (Barcelona, Spain).

### Pillow-like structures

In five graves in R12 and in 39 of Ghaba a whitish and powdery deposit was observed, usually in a very circumscribed area of the grave. Three of these deposits were investigated for opal phytoliths and the analysed phytoliths were directly radiocarbon dated [27], [28]. The analysed sample from R12 was collected from the deposit behind the skull of grave 46, associated with an early phase of the site. The two samples from Ghaba were collected from the deposits behind the skulls of graves 233 and 295. Small quantities of these pure samples were mounted directly on slides with Entellan. The analysis of phytoliths focused on the pattern of elongate cells from silica skeletons to allow as accurate as possible taxonomic identification of grasses. Phytolith samples for radiocarbon dating were processed according to [29].

### Dental calculus

Dental calculus was sampled from two skeletons from R12 and 18 skeletons from Ghaba according to standard methods [30]. All erupted deciduous and permanent tooth crowns were visually examined. Calculus was removed from teeth with large deposits and a well-preserved antimere (corresponding opposite tooth) so that a record of the calculus formation for each tooth type would be preserved. The calculus was carefully peeled away with a dental tool in sterile and starch-free conditions by applying force against the edge of the deposit, in a direction parallel to the long axis of each tooth, so as not to damage the underlying enamel. Recovered calculus fragments were placed in 1.5 ml Eppendorf tubes. The tubes were topped up with 5% sodium hexametaphosphate and stirred to deflocculate the samples. After 24 hours, samples were sonicated for 5 min. and centrifuged at 2000 rpm for 5 min. The supernatant was pipetted out. After adding distilled H_2_O to the tube this step was repeated three times. Subsequently, samples were topped up with 5% hydrochloric acid (HCl) to dissolve the calculus fragments and release plant microfossils. When no more calculus fragments were visible (after 30 to 180 minutes), samples were centrifuged at 2000 rpm for 5 min. and the supernatant pipetted out again. Samples were cleaned four times with distilled H_2_O and dried at 40°C. To prepare the slides, 20 µl of distilled H_2_O was placed in the tube and the residue suspended by agitation using the pipette point; the suspension was recovered and mounted in 50% glycerol. Powder-free gloves were worn during all phases of the process.

### Microscopic analyses

All samples were observed with a Leica DM2500 microscope equipped with a Leica DF470 camera for microphotography. Slides were scanned at 200× magnification and the observed microremains were identified and photographed at 630× magnification.

### Taxonomical identification

Identification of phytoliths and starch grains was based on a reference collection from European, African and Asian economically relevant plants, and by using pertinent literature [31], [32], [33]. The taxonomical identification of silica skeletons usually relies on the combination of morphological and morphometrical characteristics of several cell components (e.g., long and short cells) [34], [35], [36], [37]. In some cases, the identification, even at genus or species level, can rely only on one element (for example long cells) [31], [32], [38], [39]. Our identifications have been based on long cell morphology and interlocking patters as well as, when present, on short cells and papillae. The vocabulary for describing the long cells morphology and relative dimensions is from [31] and general criteria in Table S1. Furthermore, we identified single grass short cells (important for understanding the C3 vs. C4 input) to support the silica skeleton evidence.

## Results

### Pillow-like structures

Phytolith preservation is excellent at both cemeteries (no pitted surfaces and extensive articulated sheets of silicified cells), pointing to very limited taphonomic disturbance. Silica skeletons provide evidence that the deposits from these cemeteries are dominated by epidermal sheets produced in the inflorescence bracts of grasses (Poaceae) (Table 2). These sheets are composed of long cells with occasional short cells and papillae.

**Table 2:** Silica skeletons from the pillow-like structures of R12 and Ghaba cemeteries.

	R12		GHABA			
Grave	46		233		295	
	N	%	N	%	N	%
Monocotyledoneae						
Poaceae						
Panicoideae						
Paniceae						
*Brachiaria* sp.	.	.	.	.	49	29.7
*Digitaria* sp.	.	.	1	1.0	.	.
*Digitaria*/*Echinochloa*	.	.	1	1.0	.	.
*Echinochloa* sp. type a	.	.	54	52.9	12	7.3
*Echinochloa* sp. type b	.	.	1	1.0	17	10.3
*Panicum*/*Setaria*	.	.	.	.	2	1.2
Andropogoneae						
*Sorghum* sp.	.	.	2	1.9	7	4.2
Panicoideae indet.	.	.	4	3.9	43	26.1
Pooideae						
Triticeae						
*Hordeum*/*Triticum*	29	90.7	10	9.8	5	3.0
Poaceae indet.	3	9.3.	23	22.5	22	13.3
Silicified tracheids	.	.	6	5.9	8	4.8
**Total silica skeletons**	32		102		165	

**Figure 2:**
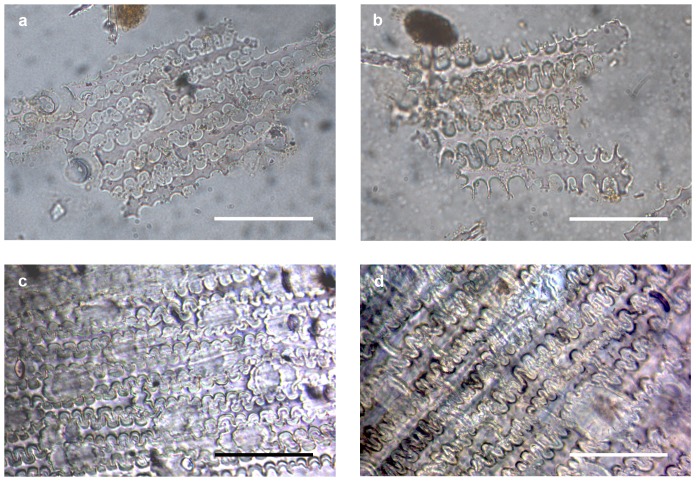
Silica skeletons from R12 compared to reference material. (**a–b**) Wheat/barley silica skeletons from grave 46, (**c**) modern silica skeleton from lemma of *Hordeum vulgare* L. and (**d**) modern silica skeleton from lemma of *Triticum turgidum* ssp. *dicoccon* (Schrank) Thell. Scale bar 50 µm.

**Figure 3:**
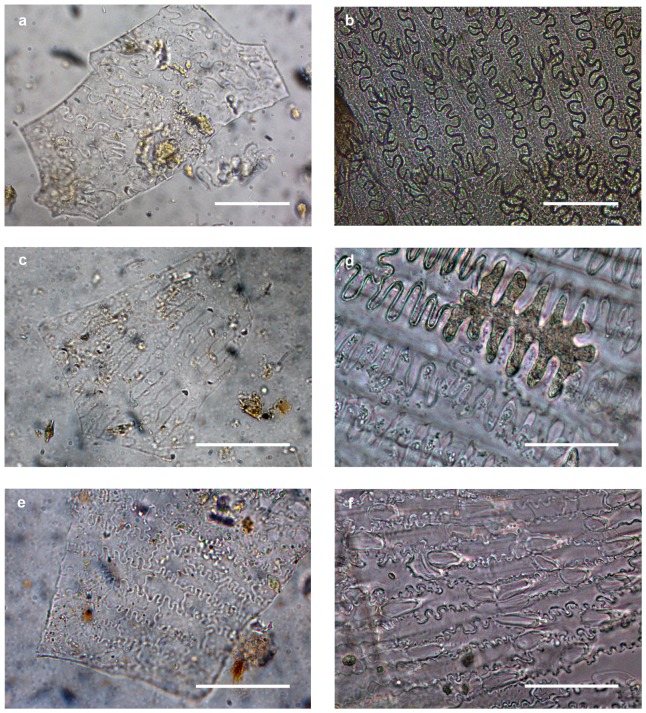
Silica skeletons from Ghaba compared to reference material. (**a**) *Brachiaria* sp. silica skeleton from grave 295, (**b**) modern silica skeleton from the inner part of the inflorescence of *Brachiaria ramosa* (L.) Stapf., (**c**) *Echinochloa* sp. *type a* silica skeleton from grave 233, (**d**) modern silica skeleton from the inner part of the inflorescence of *Echinochloa colona* (L.) Link., (**e**) *Echinochloa* sp. *type b* silica skeleton from grave 295 and (**f**) modern silica skeleton from the outer part of the inflorescence of *Echinochloa frumentacea* Link. Scale bar 50 µm. See Table S1 for the detailed description of identification characters.

The R12 assemblage is mostly comprised of silica skeletons of C3 cereals (Table 2), with dendritic long cells, several types of trapezoidal short cells and rare papillae. Morphological analysis of the material indicates its origin from plants of the wheat/barley group (*Triticum* sp. and/or *Hordeum* sp.) (Fig. 2). Moreover, the analysis of single short cells shows the predominance of rondels (92% of the short cell assemblage), further reinforcing the predominance of a pooid input – a grass subfamily that includes, among other cereals, wheat and barley. In striking contrast, the Ghaba samples are dominated by C4 grasses from different taxa, including the wide, non-taxonomic group of millets (Fig. 3). The observed silica skeletons are composed almost exclusively of long cells with very characteristic structure and side protuberances that allow identification at genus level [31] (for identification criteria see Table S1). Plants of the Paniceae tribe (Panicoideae subfamily), and particularly *Echinochloa* sp. and *Brachiaria* sp., are prevalent (Table 2). The assemblage additionally includes small quantities of *Panicum*/*Setaria*, *Digitaria* sp. and *Sorghum* sp. (Andropogonaceae tribe). Several silica skeletons with long cells with short ∩-undulated protuberances have been grouped as indeterminate Panicoideae (Table 2). These cell types tend to occur along the narrow margin of the inner parts of the inflorescence (upper lemma and palea) of several panicoid grasses as noted, for instance, in foxtail millet, common millet and green foxtail [34], [35]. Furthermore, the assemblages from Ghaba seem to have an important input from wild grasses (Poaceae indeterminate) and a small presence of wheat/barley cereals (Table 2). The analysis of single short cells from the Ghaba samples shows a predominance of panicoid morphotypes (89% of the short cell assemblage in Grave 233, and 80% in Grave 295), as well as a minor presence of chloridoids (10% in Grave 233 and 6% in Grave 295). The short cell assemblage from Grave 295 also includes pooids (6%) and other types of short cells (9%).

### Dental calculus

**Figure 4:**
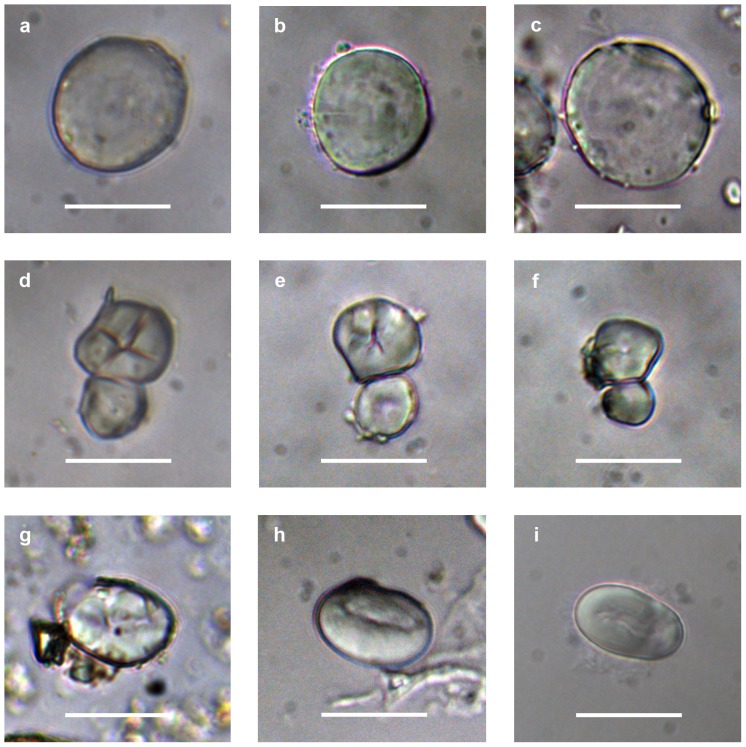
Starch grains recovered from dental calculi from Ghaba compared to reference material. (**a**) Triticeae starch grain from the skeleton of grave 169, (**b**) modern starch grain of *Triticum turgidum* ssp. *dicoccon* (Schrank) Thell., (**c**) modern starch grain of *Hordeum vulgare* L., (**d**) Panicoideae starch grains from the skeleton of grave 297, (**e**) modern starch grain of *Pennisetum glaucum* (L.) R.Br., (**f**) modern starch grain of *Sorghum bicolor* (L.) Moench., (**g**) Faboideae starch grain from the skeleton of grave 52, (**h**) modern starch grain of *Lablab purpureus* (L.) Sweet and (**i**) modern starch grain of *Vigna unguiculata* (L.) Walp. Scale bar 20 µm.

Calculus samples from Ghaba yielded primarily damaged and undamaged starch grains (N_total_ = 20). Seven large (20–40 µm) discoidal grains with smooth or rugose surface (taphonomic rugosity), and occasionally showing lamellae, are from Triticeae grasses (Fig. 4a). The identification of Triticeae starch suggests wheat and barley (Fig. 4b–c), confirming the evidence from the phytoliths. Panicoid grasses are identified by two medium-sized (10–20 µm), polyhedral, aggregated starch grains, with wrinkled surface, flat facets and centric hila (Fig. 4d). These are panicoid starch grains from big millets such as *Sorghum* or *Pennisetum* (Fig. 4e–f). Dicotyledoneae are represented by five large (20–30 µm), ovoid grains, with centric, linear hila and rugose surface (Fig. 4g). Morphological characters fall within the Faboideae subfamily of Fabaceae (legumes), which includes east African species such as the hyacinth bean (*Lablab purpureus* (L.) Sweet) and cowpea (*Vigna unguiculata* (L.) Walp.) (Fig. 4h–i). However, the taphonomised surface (rugose and without lamellae) impedes a more detailed identification. Three small to medium-sized (5–20 µm), spherical, semi-compound grains with smooth surface and centric hila (Type 1 in Table 3) were observed but no identification was possible using our reference collection. Finally, some damaged non-identifiable granules were observed (Damaged indet. in Table 3). Phytoliths were scarce in the calculus samples. An exception is grave 296, which yielded several silica skeletons of Poaceae, including panicoids. The phytolith identifications in other Ghaba dental calculus samples also saw the presence of phytoliths generally identifiable as grasses. The dental calculus from R12 yielded one Faboideae starch grain, one damaged unidentifiable starch grain and some grass phytoliths (Table 3).

**Table 3:** Plant microremains recovered from dental calculi from Ghaba and R12.

	GHABA																		R12	
Grave	52	169	195	197	199	227	231	234	248	256	257	267	275	276	278	289	296	297	71	109
**Phytoliths**																				
*Silica skeletons*																				
Panicoideae	.	.	.	.	.	.	.	.	.	.	.	.	.	.	.	1	6	.	.	.
Poaceae indet.	.	.	.	.	.	.	.	1	.	.	.	.	.	.	.	.	5	.	.	.
Total silica skeletons	.	.	.	.	.	.	.	1	.	.	.	.	.	.	.	1	11	.	.	.
*Single cells*																				
Monocotyledoneae																				
Poaceae																				
Long cells																				
Panicoideae	.	.	.	.	.	.	.	.	.	.	.	.	.	.	.	.	12	.	.	.
Pooideae	.	.	.	.	.	.	.	.	.	.	.	.	.	.	.	.	1	.	.	.
Poaceae indet.	.	.	.	.	.	.	1	1	.	2	.	1	.	.	.	.	52	.	.	1
Bulliform	.	.	.	.	.	.	.	.	.	.	.	.	.	.	.	.	4	.	.	.
Trichome	.	.	.	.	.	.	.	.	.	.	1	.	.	.	.	.	2	.	.	.
Dicotyledoneae	.	.	.	.	.	.	.	.	.	.	.	.	.	.	.	.	2	.	.	.
Indet. taxon	.	2	.	.	.	.	.	.	.	1	.	.	.	.	1	.	5	.	.	.
Total single cells	.	2	.	.	.	.	1	1	.	3	1	1	.	.	1	.	78	.	.	1
**Starch grains**																				
Triticeae	1	1	1	.	1	.	.	.	.	1	.	.	.	.	.	1	.	1	.	.
Faboideae	1	.	.	.	1	.	.	1	.	.	.	1	.	.	.	.	.	1	.	1
Panicoideae	.	.	.	.	.	.	.	.	.	.	.	.	.	.	.	.	.	2	.	.
Type 1	.	.	.	2	1	.	.	.	.	.	.	.	.	.	.	.	.	.	.	.
Damaged indet.	.	.	1	1	.	1	.	.	.	.	.	.	.	.	.	.	.	.	1	.
Total starch grains	2	1	2	3	3	1	.	1	.	1	.	1	.	.	.	1	.	4	1	1

## Discussion

The vegetal material recovered from these Neolithic Sudanese cemeteries originates from the plant exploitation strategies of human groups inhabiting the Nile Valley during the Mid-Holocene. The phytolith evidence from Ghaba's graves highlights broad spectrum exploitation of local grasses (mostly panicoids) from savannah-like environments, while the R12 sample reveals the use of cereals (wheat/barley), which probably came from the north of the Nile Valley. At the same time, the analysis of the dental calculus from several individuals from both cemeteries indicates that local grasses and domestic cereals were directly used as part of the diet of the people of Ghaba and R12. Additionally, the microfossils indicate the dietary presence of legumes and other non-identifiable sources, which might have included local tubers.

The grave samples from R12 are dominated by phytoliths from chaff of wheat/barley. The scarcity of papillae in the silica skeletons prevents a meaningful statistical analysis that would identify the remains at species level [36]. However, the wheat/barley phytoliths can be interpreted as originating from domesticated plants because their wild ancestors are missing from the region [1]. Based on published macrobotanical data from Neolithic Egypt – Merimde [1] and Fayum [2] – these finds hypothetically concern emmer wheat (*Triticum turgidum* ssp. *dicoccon* (Schrank) Thell.) and hulled barley (*Hordeum vulgare* L.). The direct date from one of the cereal phytolith concentrations (grave 46; 5311–5066 cal BC) shows that the evidence of domestic cereals from R12 pre-date the cereal finds from the Fayum and Merimde in Egypt (dated at after 4500 cal BC) and the earliest finds of barley from Kadruka 1 in Sudan (c. 4500–4000 cal BC) [40]. This implies that domesticated wheat and barley were available in regions of Egypt and Sudan at least 500 years earlier than previously thought. This confirms an earlier spread of the Near East type of agriculture towards the south. The R12 Mid-Holocene environment and river dynamics were sufficiently similar to the north Nile Valley to allow for flooding cultivation [18]. Furthermore, the early acceptance of cereals in Nubia can be attributed to people in this region being already familiar with wild grass exploitation. This probably resulted in the quick adoption of other plants, such as domesticated cereals coming from the Near East.

The phytolith samples from Ghaba, in contrast to R12, show an input from a variety of C4 grasses. This demonstrates that people at Ghaba exploited mixed stands of wild savannah grasses. Such exploitation of semi-arid ecological niches in Sudan was already suggested [41] and it is compatible with the evidence from impressions in pottery from Early Khartoum (Mesolithic) [42], Early Neolithic sites in Central Sudan [6], [7], [8] and ethnographic studies [43]. Ghaba, besides providing direct evidence of the gathering of savannah grasses, shows the considerable disparity of their use at a single site. These findings, radiocarbon dated at 5620–5480 cal BC and 4730–4540 cal BC, are not just similar to other Sudanese sites but also to the Neolithic Nabta Playa, Farafra and Abu Ballas in Egypt [44] and Mesolithic sites in Libya [15], [45], [46], [47].

The direct dietary evidence from starch grains and phytoliths found in the dental calculus confirms the result from sedimentary samples. It also shows that Ghaba has a range of plant intake covering both domestic C3 cereals and wild C4 grasses, together with legumes (Faboideae) and other non-identified starchy plants/organs. Legumes are scarcely recorded in the charred macrobotanical data [48]. In the starch assemblages from Ghaba, domestic cereals are more common than panicoid grasses, in striking contrast with the phytolith assemblages from the pillow-like structures. This pattern is most likely due to a better preservation of the starch grains of major dimensions [49], such as those of domestic cereals. The evidence from R12 is unfortunately poorer, only confirming the presence of legumes and grasses in general.

Considering all this, the evidence from the Nubian and Central Sudan Neolithic sites of R12 and Ghaba suggests broad-spectrum exploitation of plant resources by these Neolithic communities. The presence of phytolith-rich deposits advocates for the systematic use of intact grass inflorescence and/or inflorescence by-products as grave goods in Neolithic Sudan. Indeed, similar whitish deposits were observed at Neolithic WT1 Pyramid at Sedeinga, north of Kerma, and the Late Neolithic cemetery Kadada C near Ghaba [50]. The finds from both phytoliths and starch reveal new information on the diet of these human groups, indicating a diverse intake of grains (cereals, various millets and legumes). R12 is dominated by domestic Near East cereals and provides rare direct evidence of the cultivation of crop plants in a society whose economy has been considered mainly pastoral. This cemetery also provides the first evidence in Africa for the use of the near-eastern wheat/barley crop assemblage. Finally, the calculus microremains imply the consumption of green parts and/or reservoir organs from plants of savannah-like environments, including riverine areas.

## Supporting Information

Table S1 **Identification criteria for Panicoideae silica skeletons.**
(DOCX)
