# Supplementary material for: Microbotanical Evidence of Domestic Cereals in Africa 7000 Years Ago
Source: PLoS One. 2014 Oct 22;9(10):e110177. doi: 10.1371/journal.pone.0110177 (PMC4206403; doi:10.1371/journal.pone.0110177)
Supplement: Table S1 — Identification criteria for Panicoideae silica skeletons. (DOCX) [file pone.0110177.s001.docx]

**Table S1.** Identification criteria for Panicoideae silica skeletons.

| **Taxa** | **Description** |
| --- | --- |
| *Brachiaria* sp. | Long cells with lobated, simple protuberances on the sides with a length to width ratio of ca. 1:1. The main, central body is generally of a greater size than the amplitude of the protuberances. Cell edges are columellate. The silica skeletons have a lobated cell interlocking pattern. |
| *Digitaria* sp. | Long cells with simple, unbranched, deep and serrate side protuberances, of which the height is much greater than width. Cell edges are truncated. The silica skeletons have very serrated, zigzagging cell interlocking with flat or semi-flat edges between cells along the same row. |
| *Echinochloa* sp. type a | Long cells with simple or branched, columellate, deep protuberances on the sides, with the height being much greater than the width. The main, central body is generally of a smaller size than the amplitude of the protuberances. Cell edges are tuberculate or crenate. The silica skeletons have a deeply undulate cell interlocking pattern. |
| *Echinochloa* sp. type b | Long cells with deep irregular side protuberances. The protuberances vary from simple (more rarely) to branched/lobate (very common). The main, central body of the long cell is of similar or greater size than the amplitude of the protuberances. Cell edges are tuberculate or crenate. The silica skeletons have a deeply undulate cell interlocking pattern. |
| *Panicum*/*Setaria* | Long cell walls with both Ω-undulated and η-undulated margins. |
| *Sorghum* sp. | Long cells with mostly serrated, simple protuberances. The main central body of the long cell is of greater size than the amplitude of the protuberances. Cell edges are generally truncated. The silica skeletons have distinctive serrated, orthogonal zigzagging, cell interlocking pattern. |
